# Postmenopausal giant uterine adenomyoma with adipose metaplasia: A case report and literature review

**DOI:** 10.1097/MD.0000000000038885

**Published:** 2024-07-12

**Authors:** Xuemei Qing, Min Xie, Hongying Guo, Bangfang Xie, Hailong Huang, Yong Zhang, Ying Ma

**Affiliations:** a Department of Obstetrics and Gynecology, Qingbaijiang District People’s Hospital, Chengdu, Sichuan, China; b Department of Obstetrics and Gynecology, Southwest Medical University, Luzhou, Sichuan, China; c Department of Obstetrics and Gynecology, Mianyang Central Hospital, Mianyang, Sichuan, China; d Department of Obstetrics and Gynecology, Chengdu Medical College, Chengdu, Sichuan, China.

**Keywords:** adipose metaplasia, case report, giant, postmenopausal, uterine adenomyoma

## Abstract

**Rationale:**

Uterine adenomyomas (UAs) are common benign tumors, usually not exceeding 280 g or the weight of the uterus at 12 weeks gestation. Postmenopausal giant UAs of diameter larger than 20 cm are rare, as well as steatosis, but curable by surgical excision. Few cases of postmenopausal giant UAs have been reported.

**Patient concerns:**

Herein, we report a case of a 70-year-old female patient who presented with a giant pelvic tumor of about 20 cm × 18 cm × 20 cm with postmenopausal vaginal bleeding, and whose radiographic manifestations did not exclude the possibility of uterine malignancy.

**Diagnoses:**

Histopathology confirms an adenomyoma with partial adipose metaplasia.

**Interventions:**

We did an open laparotomy of hysterectomy, bi-adnexectomy, and pelvic adhesion release for the patient.

**Outcomes:**

Pathology revealed adenomyoma with adipose metaplasia. The patient recovered well and was discharged on postoperative day 7 with satisfactory follow-up.

## 1. Introduction

Adenomyosis is the invasion of endometrial stroma and glands into the myometrium of the uterus, classified into 2 main types diffuse and focal.^[1]^ The focal type of adenomyosis includes uterine adenomyoma (UA) and cystic adenomyosis. UA, a benign tumor of the uterus, commonly occurs in reproductive-age and perimenopausal women, with typical clinical manifestations of uterine enlargement, uneven thickening of the myometrial wall, or even a tumor bulge. Similar to but different from the uterine smooth muscle tumors, UA is not demarcated from the myometrium, with a not-so-large tumor size; due to their estrogen-dependent character, most tumors shrink or even disappear after menopause, while postmenopausal giant adenomyoma of more than 20 cm caliber is rare. In this article, we report a case of a 70-year-old patient admitted to our hospital in December 2023, with a giant adenomyoma and postmenopausal vaginal bleeding, combined with the radiographic manifestations, uterine malignancy was not excluded, so it was resected surgically, with final pathology confirmed a UA with partial adipose metaplasia. A literature review of previous cases summarizes the clinical features and treatment of UA.

## 2. Case presentation

**Figure 1.**
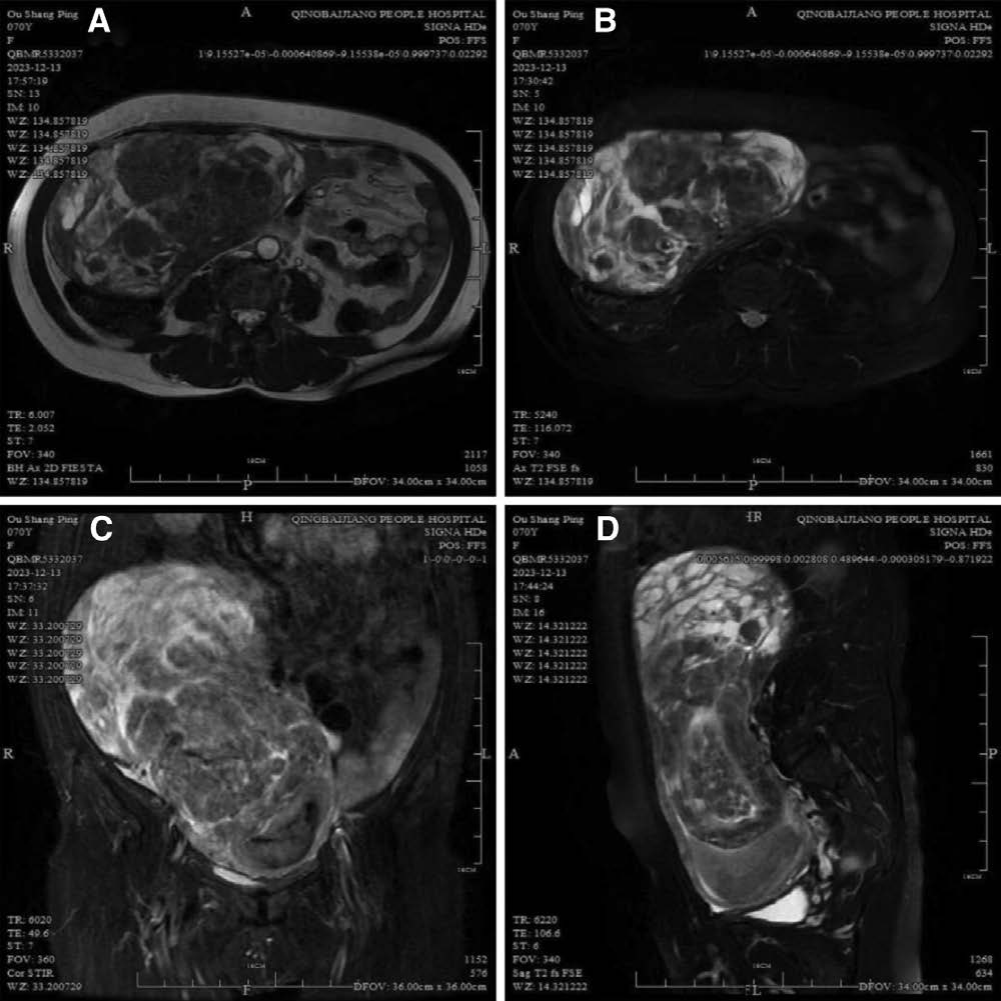
MRI imaging of a giant tumor on the right side of the abdominopelvic cavity, with some areas of high signal, which is possibly a neoplastic lesion of uterine origin or uterine sarcoma. (A) Cross-sectional image of T1-weighted image. (B) Cross-sectional image of T2. (C) Coronary image of T2. (D) Sagittal image of T2. MRI = magnetic resonance imaging.

A 70-year-old Chinese female presented to the local Community Health Center and transferred to the gynecology department of our hospital with a complaint of menopause for 15 years and vaginal bleeding for 2 months. Two months ago, she experienced unexplained vaginal bleeding that was heavier than her previous menstrual flow, dark red, with clots, and without other discomfort. Deny history of chronic diseases, infectious diseases, and allergies. G_5_P_5_ with 5 vaginal deliveries. Gynecological examination: normal vulvar development, patent vagina with a small amount of bloody fluid, atrophic cervix without columnar epithelial ectasia or heterogeneous blood vessels, a small amount of active bleeding from the uterus, the uterine corpus and both adnexa were not clear on palpation, and a giant tumor was found in the abdomen with its upper edge up to the 4th transverse finger and its right edge up to the anterior axillary line, with good mobility and no pain. At the local Community Health Center: carbohydrate antigen-125: 39.72 U/mL. No abnormalities in the remaining laboratory analyses. Gynecological ultrasound: the endometrium was unclear and moderately hyperechoic, measuring 10 cm × 5.9 cm × 10.2 cm, poorly demarcated from the myometrium, with the left edge reaching the plasma layer of the uterus in continuity. Pelvic computed tomography: a giant tumor (about 228 mm × 156.5 mm × 93 mm) was seen in the pelvis, with uneven density within the tumor. Pelvic magnetic resonance imaging (MRI) of our hospital: a giant tumor on the right side of the abdominopelvic cavity, possibly a neoplastic lesion of uterine origin or uterine sarcoma, other sources and types of neoplastic lesions must be excluded (Fig. 1A‐D).

**Figure 2.**
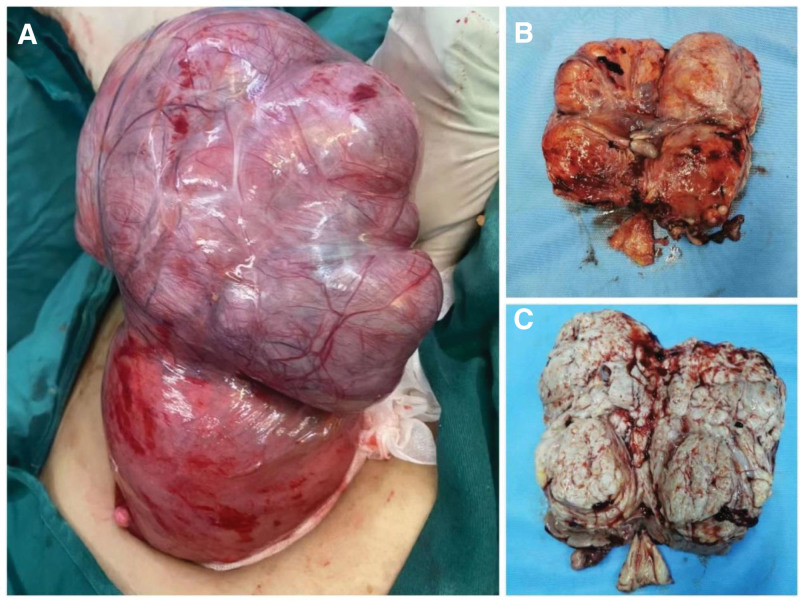
Naked eye view of the section of the isolated uterus and the tumor. (A) A tumor about 20 cm × 18 cm × 20 cm on the right side of the uterus. (B) The tumor poorly demarcated from the myometrium. (C) Grayish-yellow degenerated tissue in the tumor section.

**Figure 3.**
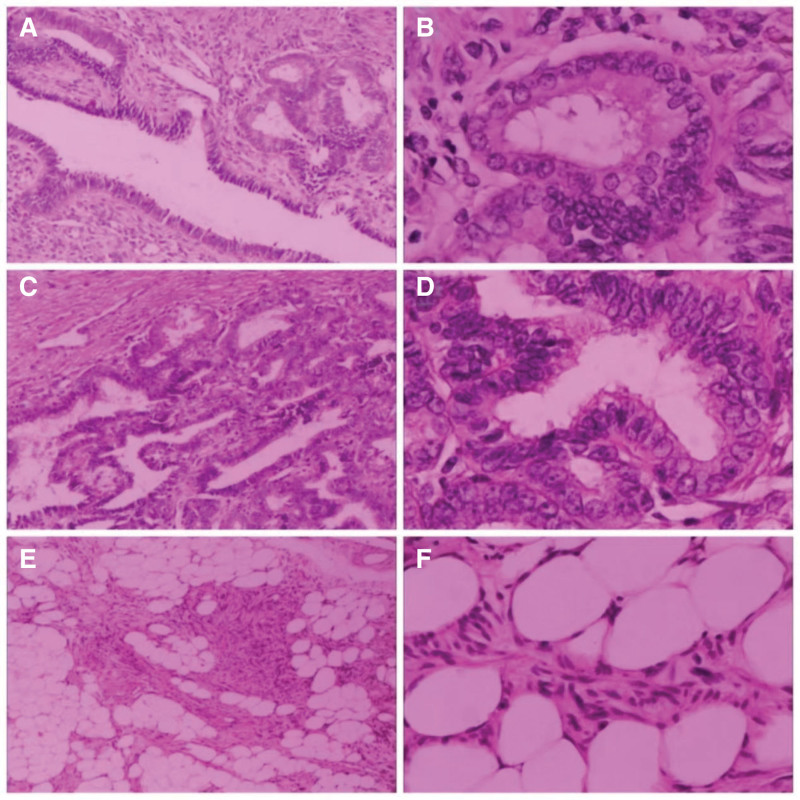
Histopathology of adenomyomas of the uterus with adipose metaplasia, Hematoxylin-Eosin staining. Endometrial glands were visible in the myometrium for (A and C) (×100), (B and D) (×400). Adipose metaplasia within adenomyoma tissue for (E) (×100) and (F) (×400).

**Figure 4.**
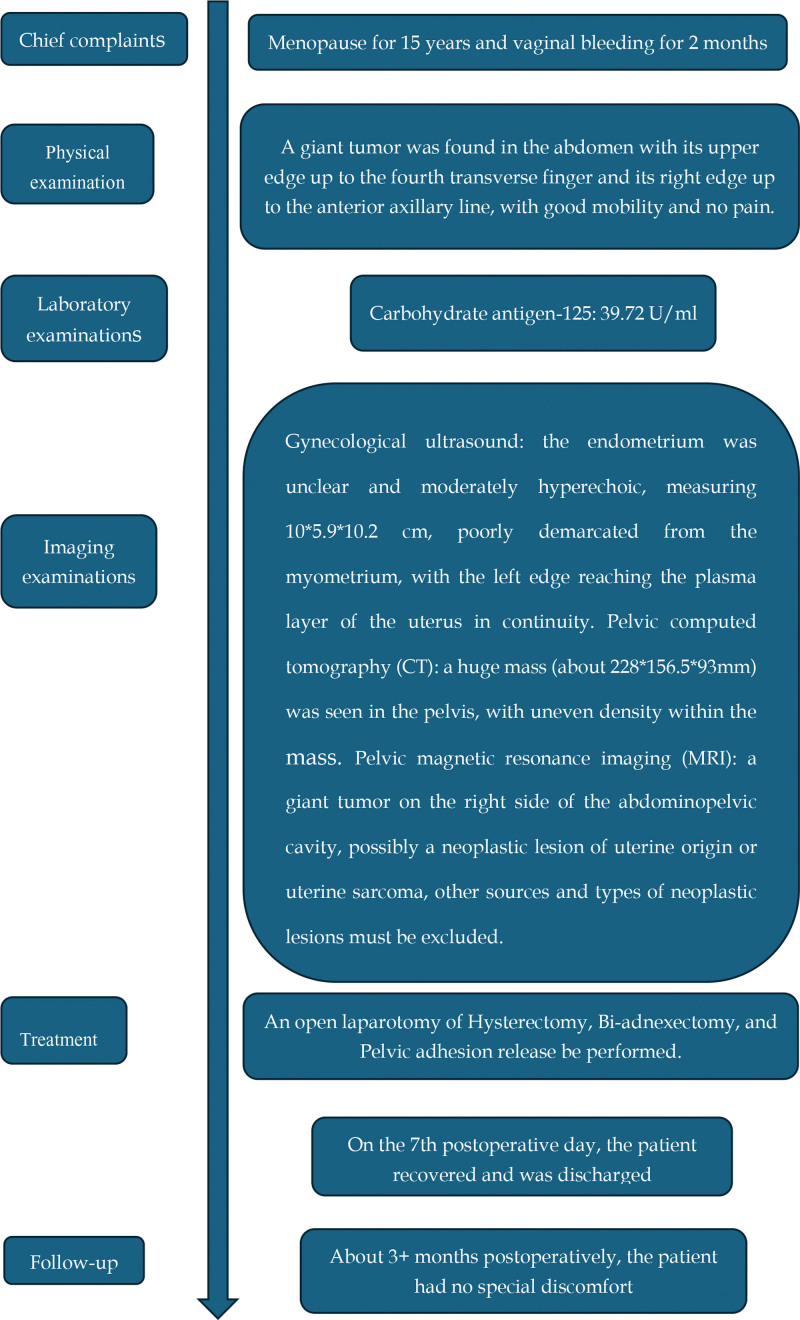
Timeline.

The patient was suspected to have a uterine tumor (possibly malignant) and then an open laparotomy of hysterectomy, bi-adnexectomy, and pelvic adhesion release was performed. Intraoperative examination: about 50 mL of yellowish fluid in the pelvis, the uterus enlarged like a pregnant woman of more than 8 months, a tumor about 20 cm × 18 cm × 20 cm on the right side of the uterine corpus (Fig. 2A), with rich blood supply, 3 tumors about 2 cm on the uterus anterior wall, the retina was adhered to the uterus posterior wall, with a normal appearance of bilateral annexes. Anatomy of isolated uterus: 3 smooth muscle tumors about 2 cm on the anterior wall, with obvious parafilm, a tumor about 20 cm × 18 cm × 20 cm on the right side, with softer texture, poorly demarcated from the myometrium (Fig. 2B), and with grayish-yellow degenerated tissue in the tumor section (Fig. 2C), 600 mL of dark red blood accumulation in the uterine cavity, the endometrium was smooth, and cervix was atrophic. Pathology confirmed adenomyoma (Fig. 3A‐D) with some areas of adipose metaplasia (Fig. 3E‐F). On the 7th postoperative day, the patient recovered and was discharged. About 3+ months postoperatively, the patient had no special discomfort. The timeline can be seen in Figure 4.

## 3. Discussion

The patient reported in this article was a 70-year-old woman with postmenopausal vaginal bleeding and a giant pelvic tumor, with imaging findings that did not exclude uterine malignancy, which was surgically excised and ultimately pathologically confirmed to be a UA with some adipose metaplasia. Typically, adenomyoma tumors tend to be small, the affected uterus often retains its overall shape, and rarely exceeds 280 g or the weight of a uterus at 12 weeks of gestation; degeneration is rare, and malignancy is even lower, about 1%, which most commonly occurs in the elderly, with the most common risk factor being long-term exposure to estrogens.^[2]^ Due to its estrogen dependence, the tumor is expected to disappear with age, with prevalence rates in postmenopausal women ranging from 2% to 4%, and postmenopausal giant UAs being rare.

Adenomyoma is a tumor formed when ectopic endometrium (glandular and mesenchymal) invades the myometrium and grows in a confined fashion, causing reactive hyperplasia of the surrounding muscle fibers and fusing with normal myometrial tissue.^[3]^ The prevalence ranges from 7% to 23%, but the incidence in the population is unclear and according to pathological statistics, it can be 20 to 30%.^[4]^ The main manifestations of adenomyomas are secondary progressive dysmenorrhea, dyspareunia, painful defecation, heavy bleeding, and menorrhagia. It can also cause painful bowel movements, heavy or prolonged menstrual periods, uneven enlargement of the uterus in the form of a sphere or limited nodular bulge, and even leading to infertility. It can also asymptomatically. It has been shown that a higher number of women with adenomyomas have never been pregnant, identified as a factor associated with infertility independently, and with other outcomes of adverse pregnancy like preeclampsia, miscarriage, and fetal growth restriction.^[5,6]^ These symptoms are not specific to adenomyomas and diagnosis needs to be made by other means. Previously diagnosed by histopathology of lumpectomy or hysterectomy specimens. Nowadays, due to the increased need to identify adenomyomas in young women and the rapid development of imaging technology, adenomyomas can be diagnosed noninvasively by transvaginal ultrasound (TVUS) or MRI. Microscopically, endometrial glands and mesenchyme are distributed in islands in the myometrium, originate mainly from the basal endometrium, are insensitive to progesterone, and are usually in the proliferative phase, with localized areas of possible secretory phase changes.

Ultrasonography is the method of first choice for the diagnosis of UAs, due to convenient, inexpensive, reproducible, and showing sonographic features corresponding to the pathological changes of adenomyosis. Based on the results of the latest improved Delphi program, the specific features are as follows: globular uterus, asymmetric thickening of the myometrial walls, hyperechogenic islands within the myometrium, transverse vascularity, fan-shaped shading, cysts within the myometrium, irregular junctional zone (JZ), interrupted JZ, and echogenic sub endometrial lines and buds.^[7]^ Doppler color flow imaging shows increased blood flow in the affected areas of the myometrium and helps to differentiate between adenomyomas and smooth muscle tumors. Both transabdominal and TVUS are available; TVUS has a sensitivity of 82.5% and specificity of 84.6%. When TVUS is not feasible, transrectal ultrasound can be used, and three-dimensional ultrasound improves visualization of the uterine cavity. MRI, which has an intuitive image, multiparameter and multiplanar imaging, and operator independence, has been increasingly used for diagnosis, staging, and dynamic monitoring of UA after medical treatment.^[8]^ On T2-weighted images, the high signal corresponds to the ectopic endometrial foci of hyperplasia, and the surrounding low signal corresponds to the smooth muscle hyperplasia of the myometrium with poorly demarcated, thickened, or deformed JZs.^[9]^ On T1-weighted images, the lesional tissue with hemorrhage is a high signal. MRI can also determine the origin of the tumor, but there are case reports of UAs appearing as uterine malignancies on MRI.^[10]^ Similar to TVUS, it has a sensitivity and specificity of 88% to 93% and 61% to 97%, respectively, however, due to limited availability as well as high cost, MRI is often used as a second-line examination. Elastography, with 2 types one based on shear waves and the other on strain elastography, may be superior to TVUS in differentiating uterine fibroids from UAs, but its sensitivity and specificity as well as intra- and inter-operator variability need to be further quantified and more studies are needed to clarify its place in the diagnosis of UAs.^[11]^

UA is similar to uterine smooth muscle tumor so the differentiation is particularly important. Some differentiation can be made by the above examination tools, but the main difference is that adenomyomas do not have a parafilm and are poorly demarcated from the surrounding uterine myometrium, making it difficult to completely remove them during surgery. It has been shown that DNA mutations in uterine smooth muscle tumors are also found in a subgroup of UAs and that driver mutations contribute to the development of some adenomyomas; however, the pathogenesis may be different, and large-scale genomic analyses are needed to elucidate the complete molecular background of UAs.^[12]^

The diagnosis of UA still requires histopathological examination, in which endometrial glands and mesenchyme are seen microscopically in an insular distribution in the myometrium, and the basal layer of ectopic endometrium usually forms a complex structure of horizontally interconnected glands, which is different from the glands in functional endometrium, which are isolated, unbranched, and longitudinally arranged. However, the gold standard for the histopathologic definition is still controversial. The diagnostic accuracy of adenomyoma has been steadily improving as unique molecular markers continue to be discovered.^[13]^ In this case, the isolated uterus was visually observed as follows: tumors of various sizes were seen, the largest being 18 cm × 20 cm, and the tumor section had a swirling appearance with unclear borders. Microscopically, glands were seen in the myometrial tissue and adipose droplets were seen in some areas, confirming adenomyoma with adipose metaplasia. Metaplasia is the adaptation of local tissues from one type of differentiated mature tissue to another mature tissue under pathological conditions, which is reversible and to some extent beneficial, but sometimes malignant. As estrogen levels gradually decline in perimenopausal women, the reproductive organs become smaller or even shrink, and estrogen-dependent diseases such as uterine smooth muscle tumors and UAs also become smaller or shrink. However, in this case, the diameter of the adenomyoma was more than 20 cm, and the microscopic finding of adenomyoma with some adipose metaplasia confirmed the adaptive process of local tissue transformation from adenomyoma to adipose.

UA is an estrogen-dependent disease, and medication by controlling the hormonal environment has become the first-line treatment, but recurrence is inevitable after stopping the medication, so the adaptive groups of medication are patients with adenomyoma who wish to preserve the uterus and have the desire to conceive, who are approaching menopause, or who are contraindicated for surgery. Medical therapy includes progesterone, combined oral contraceptives, dienogest, gonadotropin-releasing hormone agonists (GnRH-a), GnRH antagonists, levonorgestrel-releasing intrauterine system, danazol, and some experimental medications like antiplatelet agents, aromatase inhibitors, and oxytocin antagonists. Percutaneous microwave ablation under ultrasound and dynamic MRI for the treatment of UAs has also been popularized for clinical use.^[14]^ For patients unsuitable for long-term medical therapy, refractory adenomyosis, or those with giant adenomyomas, surgery is a viable option, with partial and complete excision of the lesion if the uterus is preserved, but with the possibility of recurrence. In the setting of high preoperative serum carbohydrate antigen-125 levels and concomitant endometriosis, postoperative administration of GnRH-a therapy reduces the risk of recurrence.^[15]^ Combination therapy may also be considered: laparoscopy, GnRH-a therapy, assisted reproductive technology, and placement of levonorgestrel-releasing intrauterine system. For cystic adenomyosis that is submucosal or near the mucosa, hysteroscopic surgery may also be performed.^[16,17]^ If all fail or there is no desire to preserve the uterus, total hysterectomy may be indicated, as recurrence of adenomyomas has been reported with subtotal hysterectomy with preservation of the cervix.^[18]^ In this case, the patient was an elderly woman with postmenopausal vaginal bleeding and a giant pelvic tumor of unknown character, and surgical exploration to clarify the diagnosis was performed at the same time as surgical treatment. Due to the intraoperative presentation of a giant UA combined and 3 smooth muscle tumors with pelvic adhesions, a surgery of hysterectomy, bi-adnexectomy, and pelvic adhesion release were performed.

In conclusion, UA is a benign tumor, and malignant transformation is rare, as is postmenopausal giant adenomyoma. In elderly women with postmenopausal vaginal bleeding accompanied by a giant pelvic tumor, the imaging with a mixed echogenic tumor suspects it as a malignant tumor. Nevertheless, it cannot be easily diagnosed before surgery, surgical resection is still necessary to clarify the diagnosis and ultimately based on histopathology.

## Acknowledgments

All authors are thanked for their contributions to the manuscript.

## Author contributions

**Conceptualization:** Xuemei Qing, Hongying Guo, Ying Ma.

**Data curation:** Xuemei Qing, Min Xie, Bangfang Xie, Hailong Huang.

**Supervision:** Xuemei Qing, Yong Zhang, Ying Ma.

**Validation:** Xuemei Qing, Ying Ma.

**Writing – original draft:** Xuemei Qing.

**Writing – review & editing:** Xuemei Qing, Min Xie, Hongying Guo, Bangfang Xie, Hailong Huang, Yong Zhang, Ying Ma.
